# Standardized evaluation of the quality and persuasiveness of mobile health applications for diabetes management

**DOI:** 10.1038/s41598-022-07544-2

**Published:** 2022-03-07

**Authors:** A. Geirhos, M. Stephan, M. Wehrle, C. Mack, E.-M. Messner, A. Schmitt, H. Baumeister, Y. Terhorst, L. B. Sander

**Affiliations:** 1 Department of Clinical Psychology and Psychotherapy, Institute of Psychology and Education, Faculty of Engineering, Computer Science and Psychology, Ulm University, Ulm, Germany; 2 Department of Rehabilitation Psychology and Psychotherapy, Albert-Ludwigs University Freiburg, Freiburg, Germany; 3 Research Institute of the Diabetes Academy Mergentheim, Diabetes Center Mergentheim (DZM), Bad Mergentheim, Germany

**Keywords:** Psychology, Endocrinology

## Abstract

This study evaluates diabetes self-management mobile health applications available from European app stores with respect to quality, concordance with recommended self-management tasks and implementation of persuasive system design principles. The European Play Store and Apple App Store were systematically searched and relevant apps were tested. Two raters independently assessed app quality using the Mobile Application Rating Scale and conducted a content analysis of provided persuasive system design principles and self-management tasks. A total of 2,269 mobile health applications were identified and 120 could be included in the evaluation. The overall quality was rated as moderate *M* = 3.20 (*SD* = 0.39, min = 2.31, max = 4.62), with shortcomings in the subcategories of engagement (*M* = 2.80, *SD* = 0.67) and information quality (*M* = 2.26, *SD* = 0.48). Scientific evidence is available for 8% of the apps. The reviewed apps implemented a median of three persuasive system design principles (range 0–15) and targeted a median of 4.5 (range 1–8) self-management tasks, however, with a lack of information about psychosocial coping strategies. Most available diabetes self-management apps lack a scientific evidence base. Persuasive system design features are underrepresented and may form a promising tool to improve app quality. Furthermore, the interaction of physical and behavioral health should be improved in existing diabetes self-management mobile health applications.

## Introduction

In Europe, about 60 million people are diagnosed with diabetes, and the prevalence and incidence rates are rising^1,2^. Diabetes care requires the performance of multiple essential self-management tasks by affected individuals. A best possible performance of diabetes self-management task, as recommended by the American Association of Diabetes Educators^3^, is decisive for the course of diabetes including lower risks of acute and long-term complications, significantly lower morbidity and mortality and higher mental well-being and quality of life^4^. Accordingly, diabetes self-management education and support are key elements for the successful self-management of the condition^5^. However, self-management education and support may be underutilized in diabetes care^3^, thus applications supporting people to better self-manage their diabetes may be useful. Mobile health applications (MHA) have been found to be a promising technological approach to help people perform better self-managements^3,6–10^. Studies support that MHA might have positive impacts on quality of life, diabetes outcomes and patient-provider communication^6,11^. In addition, MHA offer a low-threshold, cost-effective and flexible support opportunity in the everyday lives of users^12^.

Due to the often unregulated and rapidly growing nature of app stores, numerous commercial diabetes MHA are available. Since user ratings do not constitute a reliable or valid indicator of app quality^13^, it is important to systematically evaluate the quality of the available MHA in order to support health care providers, educators and users in their search for appropriate and secure apps^13,14^. For this purpose, several studies have been conducted in recent years^13,15,16^.

Hood et al.^13^ conducted a meta-review of international available MHA quality rating studies published between 2010 and 2014. Overall, the results indicate security concerns, lack of content founded upon validated theories, deficient educational information and limited implementation of behavior change techniques^13^.

In more recent quality rating studies, Gong et al.^16^ and Chavez et al.^17^ assessed the quality of diabetes self-management MHA in the largest app stores in China and the US, respectively, using the mobile application rating scale (MARS), a reliable and valid measurement of MHA quality^18^. Both studies reported suboptimal overall quality of the MHA, with the information and engagement domains scoring the worst.

To optimize engagement with MHA it is crucial to leverage technical capabilities^19,20^. The framework of the persuasive system design (PSD) model encompasses various technical principles to optimize human–machine interaction to support users in achieving their personal target behavior, thereby influencing their attitudes and behavior^21,22^. The positive impact of PSD on adherence to and effectiveness of MHA has been demonstrated in various studies^19,23,24^. Previously reported technical features in diabetes MHA include disease-related reminders, social networking features, feedback on self-monitoring and the possibility of sharing data with health care providers, with self-monitoring being the most frequently implemented feature^13,25^.

The findings by Hood et al. suggesting a substantial lack of educational information are of paramount concern as users are exposed to the risk of misinformation. Since clinical studies on the usefulness or effectiveness are often not available, the recommendations should, as a minimum standard, follow established clinical guidelines^4,5^. In this context, it is important that the focus of education is not limited to individual aspects of self-management. Areas such as coping and problem solving should also be targeted^26^.

Therefore, this study aims to systematically search and evaluate diabetes self-management MHA which are available in the European commercial app stores in English or German language to answer the following research questions:

1. What is the quality rating according to the MARS of diabetes self-management MHA available in European commercial app stores in terms of engagement, functionality, aesthetics and information?
2. Which persuasive system design features do diabetes self-management MHA include?
3. Do persuasive system design features predict the quality rating of diabetes self-management MHA?
4. Which of the self-management tasks recommended by the American Association of Diabetes Educators do diabetes self-management MHA address?

## Materials and methods

### Search strategy

The search was limited until December 2020, we searched the European Google Play Store and the Apple App Store using diabetes-related terms via a web-crawler of the Mobile Health App Database (MHAD) project^27^. This approach has been evaluated in several previous studies [e.g.^28–30^]. MHA were screened and downloaded if they (1) addressed people with diabetes or parents of children with diabetes; (2) contained educational or supporting information on diabetes self-management; (3) were available in English or German language. MHA were eligible for inclusion if they were accessible and enabled assessment. In the case of MHA which required further information for access (e.g., login data provided by physician), MHA developers were contacted and asked for access. Dead links were retrieved three times during a period of two weeks before final exclusion.

### Quality rating

Two independent raters assessed MHA quality using the German version of the Mobile App Rating Scale (MARS-G;^18,31^). All reviewers were graduates of clinical and health psychology supervised by a licensed psychotherapist with extensive psycho-somatic expertise. Before the reviewing process, reviewers underwent an online training for using the MARS. To capture interrater reliability between reviewers, initially five MHA were rated by all four reviewers, and the ratings were compared. Interrater reliability in both reviewer teams was excellent (Team 1: 2-way mixed ICC = 0.94, 95% CI 0.94 to 0.95; Team 2: 2-way mixed ICC = 0.88, 95% CI 0.81 to 0.92). Through the MARS, MHA quality can be evaluated using four subscales: 1) engagement (five items: fun, interest, individual adaptability, interactivity, target group); 2) functionality (four items: performance, usability, navigation, gestural design); 3) aesthetics (three items: layout, graphics, visual appeal); and 4) information quality (seven items: accuracy of app description, goals, quality of information, quantity of information, quality of visual information, credibility, evidence base). Each item can be rated on a 5-point scale. The MARS sum score is determined from the four subscales^18^.

The MARS sum score showed excellent psychometric properties (ICC [Intra Class Correlation] = 0.82, 95% CI: 0.81 to 0.82; internal consistency: ω = 0.93)^32^. The four subcategories demonstrated acceptable to excellent internal consistencies (ω = 0.79 to 0.90)^32^. In accordance with the MARS, three further categories were assessed: (5) therapeutic gain (four items: gain for patients, gain for therapists, risks and side effects, ease of implementation into routine healthcare); (6) subjective quality (four items: recommendation, frequency of use, willingness to pay, overall star rating); and (7) perceived impact (six items: awareness, knowledge, attitudes, intention to change, help seeking, behavioral change).

### User rating

Ratings of user satisfaction with the MHA (from 1 to 5 “stars”, higher score = higher satisfaction), which were available in the app stores, were extracted for comparison with the quality ratings according to the MARS.

### General characteristics

The description section of MARS was modified to collect the following information for each MHA: (1) app name; (2) platform; (3) language; (4) specific target group (if any); (5) cost; (6) technical aspects of potential tracking features (export possibilities; manual tracking; CGM connection); (7) data protection and privacy; (8) user rating; and (9) available scientific studies. Irrespective of whether relevant studies were referenced in the MHA app store descriptions, we searched the app developers’ websites and Google Scholar to identify available studies for each MHA.

### Persuasive system design features

We assessed the included MHA for 24 of the 28 PSD features as recommended by Oinas-Kukkonen and Harjumaa^21^. The features are divided into four subcategories: (1) primary task support; (2) dialogue support; (3) social support; and (4) system credibility. We focused on design features provided through the technical system itself. Therefore, we excluded the principle of liking (subcategory dialogue support), surface credibility, expertise, trustworthiness of content (subcategory trustworthiness). Principles were defined according to Oinas-Kukkonen and Harjumaa^21^ and Kelders et al.^19^. Examples for each principle are incorporated in Table 3.

### Concordance with recommended self-management tasks

The American Association of Diabetes Educators recommends eight tasks as important for successful diabetes self-management and education^3^: (1) diabetes pathophysiology and treatment options; (2) healthy nutrition; (3) physical activity; (4) medication usage; (5) monitoring; (6) preventing, detecting and treating acute and chronic complications; (7) healthy coping with psychosocial issues and concerns; and (8) problem solving. We conducted a content analysis and systematically rated which of these tasks were addressed in each MHA’s educational content. Educational content could be delivered as text, video/audio, games, or forums. We calculated the median number of self-management tasks addressed by each MHA’s content.

### Statistical analyses

Means (M) and standard deviations (SD) for the MARS sum score and all subscales were calculated. Frequency of implemented general characteristics, PSD principles and self-management tasks are reported. Furthermore, bivariate correlations and hierarchical regression analysis were performed to evaluate a potential association between PSD subcategories and MARS sum score or subcategories. Additionally, correlations between user ratings and the MARS scales scores were estimated.

## Results

### Search

**Figure 1:**
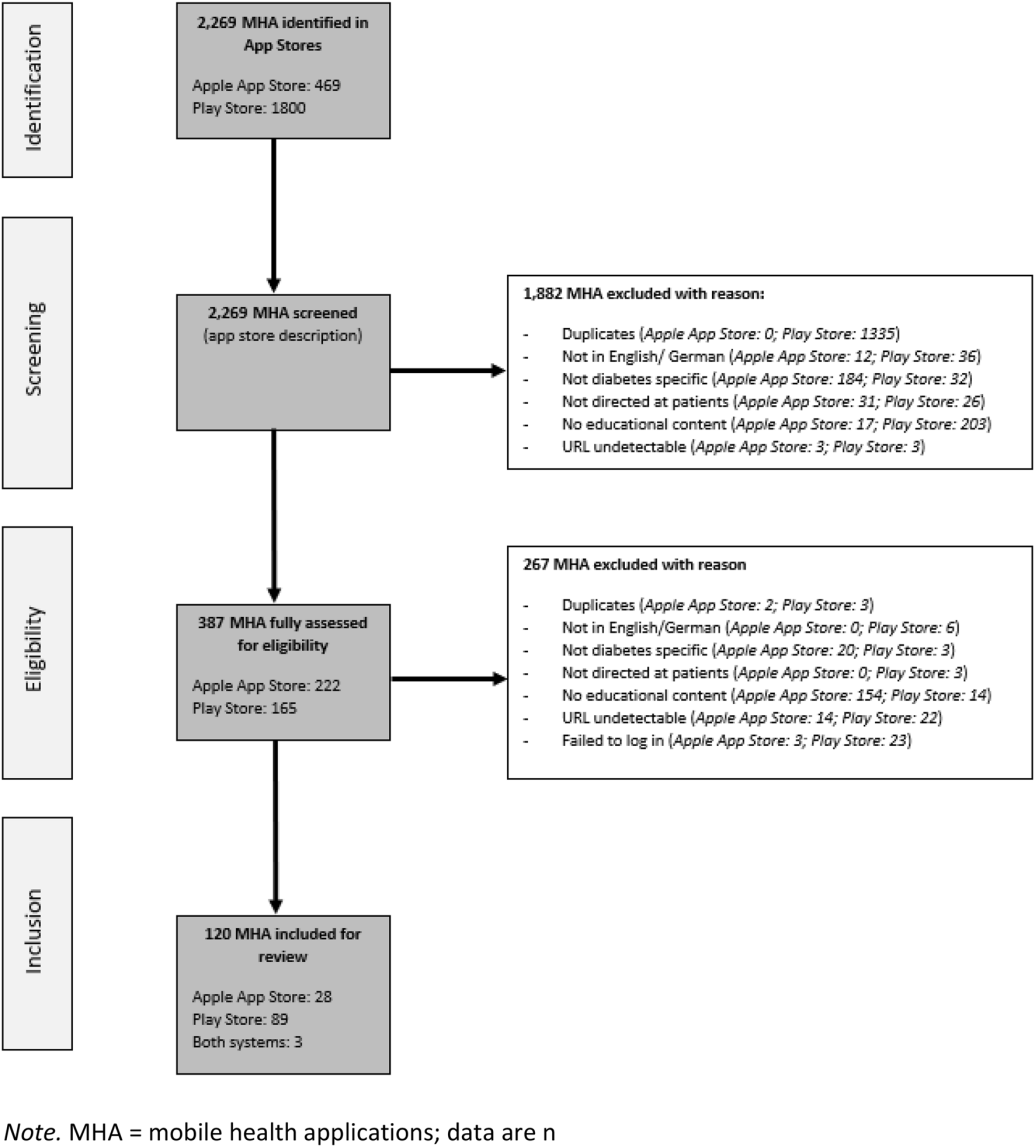
Flow-chart showing selection of MHA.

120 MHA were finally included in the app rating. Of these, 89 (74%) were available in the Play Store, 28 (23%) in the Apple App Store and three (3%) in both systems (Fig. 1).

### General characteristics

Thirteen of the included MHA (10.8%) specifically targeted type 1 diabetes, eight (6.7%) type 2 diabetes and two (1.7%) gestational diabetes. Type of diabetes was not specified for 97 (80.8%) of the MHA. One hundred and three MHA (85.8%) were available free of charge. The fees for the 14.2% of MHA charging fees varied between 0.59€ and 64.99€. Two of the MHA (“myDiabetes”, “X-Pert”) could only be accessed via a code to be provided by the user’s treating physician. All 120 MHA were available in English, however, 22 (18.3%) of them were additionally available in German. Nine of the MHA (7.5%) were designed for parents of children with Type 1 Diabetes (“DIABETES TREATEMENT App”; “Diabetes Type 1”; “Diabetes Care”; “Diabetes treatment”; “Diabetes–Diabetes Diet Tips”; “Hypo Program”; “Broteinheiten”; “KE-Finder”; “Our Journey with Diabetes”). In addition, three of the MHA were specifically designed for children (“Jerry the Bear”) and/or adolescents and young adults with diabetes (“Invincible”; “Kids and Teens Diabetes”). Regarding user privacy and data security, ten of the MHA (8.3%) were password-protected, 20 (16.7%) had a login page, 96 (80.0%) provided information on a privacy policy and 119 (99.2%) incorporated a contact or legal notice. Furthermore, 35 of the MHA (29.2%) offered a tracking feature. Among these, 23 (19.2%) offered manual tracking, while twelve (10.0%) offered automatic tracking by connecting to a CGM system; a function to export tracking data was available in 17 MHA. With regard to the evidence base of MHA, seven of the MHA (5.8%) (“Diabetes Forum”; “Hypo Program”; “myDiabetes”; “Dario”; “RapidCalc Diabetes Manager”; “Diabetes Tracker by MyNetDiary”; “iHealth Gluco Smart”;^33–36^) had been evaluated in usability or non-controlled studies and two of them (2%; “One Drop Diabetes Management”; “X-Pert”;^37,38^) in randomized controlled trials.

### Quality rating

The MARS subcategory functionality revealed the highest scores (*M* = 4.10, *SD* = 0.30), followed by aesthetics (*M* = 3.64, *SD* = 0.50) and engagement (*M* = 2.80, *SD* = 0.67). Information quality had the lowest rating scores (*M* = 2.26, *SD* = 0.48). Detailed information on the MARS sum scores and subcategory scores for the ten best-rated MHA per app store are given in Table 1. MARS ratings for all reviewed MHA are reported in Supplementary Table S1.

**Table 1:** MARS ratings for the ten highest ranking apps per app store in descending order (MARS ratings for all 120 apps in Supplementary Table S1).

Name	Developer	Total score	Quality rating				Additional subscales		
			Engagement	Functionality	Aesthetics	Information Quality	Therapeutic Gain	Subjective Quality	Perceived Impact
**Available from Play Store**									
myDiabetes*	my mhealth	4.62	4.80	4.75	5.00	3.93	4.75	4.38	4.58
Invincible *	Invincible Corp	4.02	4.30	4.38	4.84	2.58	2.25	3.63	3.75
BD Diabetes Care App	BD-Digital Health	4.01	4.20	4.38	4.67	2.79	2.25	3.75	2.50
Glucose Buddy Diabetes Tracker *	Azumio Inc	3.95	4.3	4.25	4.17	3.07	2.50	3.88	3.92
Hypo Program	Diabetes Digital Media	3.90	3.80	4.38	4.34	3.08	2.13	3.38	2.42
Jerry the Bear	Sproutel. Inc	3.88	3.80	4.25	4.67	2.79	2.00	3.13	2.25
GLUCOSEZONE	Fitscript	3.82	4.40	3.75	4.00	3.15	2.38	3.75	3.58
Diabetes Words	LES LABORATOIRES SERVIER	3.74	3.30	4.38	4.84	2.43	2.13	3.38	2.50
DiabTrend-Diabetes Assistant	DiabTrend AI Analytics Kft	3.71	4.10	4.25	4.00	2.50	2.50	3.50	3.25
My DiabetesConnect	Dr Ryzian Nizar MD MRCPUK	3.65	3.60	4.38	3.84	2.79	2.13	3.00	3.42
**Available from Apple App Store**									
myDiabetes*	My mhealth	4.62	4.80	4.75	5.00	3.93	4.75	4.38	4.58
X-PERT	Pulse Digital	4.61	4.60	4.50	4.84	4.50	4.13	4.38	4.67
Invincible *	Invincible Corp	4.02	4.30	4.38	4.84	2.58	2.25	3.63	3.75
Glucose Buddy Diabetes Tracker*	Azumio Inc	3.95	4.3	4.25	4.17	3.07	2.50	3.88	3.92
One Drop Diabetes Management	Informed Data Systems. Inc	3.93	4.00	4.38	4.00	3.36	2.88	3.50	3.75
Dario	LabStyle Innovation Ltd	3.78	4.00	4.25	4.17	2.72	3.13	3.13	3.42
Diabetes Tracker by MyNetDiary	MyNetDiary Inc	3.70	3.80	4.13	4.17	2.72	2.25	2.75	3.25
iHealth Gluco Smart	iHealth Labs Inc	3.67	3.90	4.00	3.84	2.93	2.38	3.50	3.08
Diabetes App: BD Diabetes Care	Becton. Dickinson. and Company	3.62	3.60	4.25	4.00	2.64	2.25	3.50	3.67
DMP	TLC Platforms Inc	3.61	4.00	4.00	4.17	2.29	1.88	3.50	3.09

*MHA rated in both systems; numbers represent mean score.

### User rating

Sixty-two (51.7%) of MHA had been rated by users at the time of review. The mean user satisfaction rating was 4.14 points on a five-point scale (*SD* = 0.74). Forty-four (71%) MHA received a mean satisfaction rating of 4 points or higher. There was a significant correlation between the user ratings and the MARS sum scores (*r* = 0.31, *p* = 0.015) as well as between the user ratings and the subcategory functionality (*r* = 0.37, *p* = 0.003). No significant correlations with further subcategories were found (information quality: *r* = 0.23; *p* = 0.070; engagement: *r* = 0.23, *p* = 0.073; aesthetics: *r* = 0.22, *p* = 0.081).

### Implementation of persuasive system design features

A median of three PSD principles were implemented in the MHA. The most frequently implemented principles were: system credibility (*M* = 1.62, *SD* = 0.87), dialogue support (*M* = 1.42, *SD* = 0.98), primary task support (*M* = 0.83, *SD* = 1.25) and social support (*M* = 0.27, *SD* = 0.69). Table 2 shows how many principles of each PSD category were implemented per MHA. Table 3 provides the percentages of the MHA incorporating a particular PSD principle. The MARS sum score showed significant correlations with system credibility (*r* = 0.23, *p* = 0.010), dialogue support (*r* = 0.64, *p* < 0.001), primary task support (*r* = 0.54, *p* < 0.001) and social support (*r* = 0.41, *p* < 0.001). Dialogue support and social support significantly predicted the MARS sum score. Overall, PSD subcategories can explain a significant proportion of variance in the MARS sum score (R^2^ = 0.44, *p* < 0.001; see Table 4). PSD principles per MHA are shown in detail in Supplementary Table S2.

**Table 2:** Proportion of apps implementing principles of one of the four persuasive system design categories.

	Number of implemented principles					
	0	1	2	3	4	5
System credibility (%)	1.7	56.7	22.5	16.7	2.5	–
Dialogue support (%)	11.7	55.0	17.5	11.7	4.2	–
Primary task support (%)	59.2	16.7	13.3	5	4.2	1.7
Social support (%)	81.7	12.5	3.3	1.7	0.8	–

Numbers represent how many apps implemented which amount of the seven principles of each persuasive system design category.

**Table 3:** Number of apps incorporating a particular PSD principle.

Implemented principles (%)		Example
**System credibility**		
Real-world feel	98.3	Providing contact data of developers
Authority	14.2	Citations of clinicians
Third-party endorsements	28.3	Recommendations of diabetes institutions
Verifiability	22.5	Providing source of information and links to it
**Dialogue support**		
Praise	23.3	Feedback/compliments on tracked data
Rewards	2.5	Collecting stars for engaging in lessons
Reminders	16.7	System based daily pop-up messages
Suggestion	85.8	Recipes for healthy nutrition
Similarity	4.2	Stories of other diabetes patients
Social role	7.5	Guidance by an avatar
**Primary task support**		
Reduction	16.7	Dividing tracking in small simple steps
Tunneling	6.7	Implementing sequential lessons
Tailoring	25.0	Adapting information according to type of diabetes
Personalization	10.0	Possibility to customize interface
Self-monitoring	28.3	Feature for glucose tracking
Simulation	0.8	Calculator how glucose level changes during the day
Rehearsal	5.0	Knowledge quiz
**Social support**		
Social learning	2.5	Board showing activity of other app users
Social comparison	0	Comparison of user’s response to others’ responses
Normative influence	13.3	Comparing glucose values to healthy peers
Social facilitation	7.5	Online discussion forum
Cooperation	1.7	Giving advice in small peer groups
Competition	0	Leader boards on daily activity level
Recognition	7.5	Liking and posting achievements

System credibility, dialogue support, primary task support and social support are the four categories of persuasive system design. Each category consists of seven principles.

**Table 4:** Hierarchical regression analysis for persuasive system design categories predicting MARS sum score.

	Model 1			Model 2			Model 3			Model 4		
Variable	*B*	SE *B*	*β*	*B*	SE *B*	*β*	*B*	SE *B*	*β*	*B*	SE *B*	*β*
Dialogue support	0.26	0.03	0.64***	0.22	0.04	0.55***	0.21	0.04	0.52***	0.20	0.04	0.50***
Primary task support				0.04	0.04	0.12	0.02	0.03	0.07	0.02	0.03	0.06
Social support							0.11	0.04	0.20*	0.11	0.04	0.20*
System credibility										0.04	0.03	0.10
R^2^	.41			.41			.44			.44		
∆R^2^				0.000			0.028			0.004		

***p < 0.001; **p < 0.01; *p < 0.05; B, unstandardized regression coefficient; *β*, standardized regression coefficient.

### Concordance with recommended self-management tasks

The evaluated MHA addressed a median of 4.5 of the eight recommended self-management tasks. Ninety-three MHA (77.5%) provided educational content on pathophysiology of and treatment options for diabetes. Furthermore, healthy nutrition (n = 92, 77%), physical activity (n = 79, 66%), monitoring (n = 67, 56%), as well as preventing, detecting and treating acute and chronic complications (n = 69, 58%) were frequently addressed. Problem solving (n = 31, 26%), medication usage (n = 45, 38%) as well as healthy coping with psychosocial issues and concerns (n = 44, 37%) were less often addressed. Twenty-four MHA (20%) targeted 1–2 tasks, 36 (30%) 3–4 tasks, 38 (32%) 5–6 tasks and 15 (12%) seven tasks; all eight self-management tasks were addressed in seven MHA (6%). A detailed overview of the implemented self-management tasks in each MHA is provided in Supplementary Table S3.

## Discussion

In this systematic evaluation of 120 commercially available diabetes self-management MHA, we observed an average overall quality (MARS rating) of the MHA (*M* = 3.20, *SD* = 0.39) with a median of three implemented PSD principles per MHA. Notably, implemented PSD principles predict MHA quality ratings and the potential of PSD is not exploited yet.

Previous studies on quality using MARS of US-American (*M* = 2.99, *SD* = 0.64)^17^ and Chinese diabetes MHA (*M* = 3.42, *SD* = 0.66)^16^ yielded similar results to our study. Notably, in this study the MARS sum score as well as the subscale functionality were correlated significantly with the user ratings. This is in contradiction to previous evaluations reporting that MARS ratings were not correlated with user ratings^28–30,39,40^. Given that users commonly rely on user ratings when choosing a MHA for download^41^, it is an encouraging finding that there are correlations between user ratings and MARS quality ratings in the context of diabetes self-management MHA.

In line with previous studies, the reviewed MHA had higher scores in the functionality and aesthetics subcategories, but lower scores in the engagement and information quality domains^16,17^. A potential solution to improve engagement is to implement PSD features. The concept of PSD was taken into account in the development of the MARS^18^ and our results support that the PSD principles of dialogue support and social support, in particular, predict MHA quality ratings and may strengthen the perceived quality of the MHA.

According to the present findings, the least commonly implemented PSD features are social support features. Peer support has been shown to be highly relevant for reducing diabetes distress, diabetes self-care and clinical outcomes, such as mortality^42–44^. Although online discussion forums are the most frequently implemented social support feature in the MHA examined in the present study, this type of support is often associated with ethical concerns. For instance, there may be counterproductive exchanges between individuals or the anonymity of individuals may not be guaranteed^45^. Research on the effectiveness of alternative social support principles, which could avoid some of these issues, is still limited and new approaches of social support in MHA should be evaluated^46^. A further strategy could be to implement multimodal support. For example, the use of MHA could be complemented by telehealth support by peers or clinical staff members^47^. Notably, the highest rating MHA in this study allows interaction with clinical staff members, suggesting different possibilities of personal interaction.

Regarding the PSD principle dialogue support, we found reminders and suggestions to be the most commonly used principles. Reminders, in particular, seem to be a key app component for supporting successful diabetes management^48^. An overarching aim of dialogue support features is enhancing the users’ impression that the system is a real person. This can be maintained through the use of avatars which guide the user through the MHA^49^. Recent studies demonstrated that a virtual avatar providing diabetes treatment information could improve the users’ diabetes self-management^50,51^.

Principles of system credibility features are widely implemented in the reviewed MHA by providing contact information of MHA developers. In this respect, other principles could be exploited to a greater extent. For instance, incorporating authority figures or offering opportunities for verification (e.g., literature links) could encourage users to perceive the information as more credible and could therefore increase their engagement^52^.

Finally, in terms of primary task support principles, self-monitoring was found to be most commonly addressed (e.g., apps requesting regular entries of measured glucose values). However, manual entries can be demanding for people with diabetes. Especially for people living with type 1 diabetes, the compactness and ease of use of glucose monitoring could be improved by implementation of an automated MHA-to-sensor connection. Yet, only 10% of evaluated MHA offered this option. Based on the technological possibilities, improvements should be made to facilitate glucose monitoring options^53^. Another crucial principle of primary task support is tailoring the content to people with diabetes’ needs^54^. MHA content regulation by diabetes care providers could be an innovative and effective approach to this. For instance, some MHA can be directly used for physician–patient interaction and treatment adjustment wherein the treating physician can set and change the treated person’s goals based on the monitored data (e.g., “myDiabetes”). Tailoring may also be particularly important with regard to age appropriateness (e.g., MHA appealing to younger people). With only three MHA targeting the needs of children and adolescents, the present study findings suggest major shortcomings in this area. Children are only able to understand and comply with self-management recommendations if the provided information is appropriate to their level of cognitive development^55^. Furthermore, children and adolescents are confronted with age-specific disease-related concerns (e.g., peer-group and diabetes, alcohol and diabetes), for which appropriate content need to be established^56,57^. Devising age-appropriate MHA could thus help improve diabetes-related outcomes particularly in children and adolescents^58,59^.

Aside from the technical support by MHA through PSD, this review also focused on the incorporated self-management tasks. The median of 4.5 targeted self-management tasks per MHA suggests significant improvements in the content of diabetes self-management MHA since the findings of Hood et al.^13^, who reported that MHA contained too little educational information. However, the self-management tasks of problem solving and coping with psychosocial issues are only addressed in one third of the MHA. Given the great importance of mental health in people with diabetes and the lack of time in routine clinical care for discussing mental health problems with concerned persons^60–62^, eHealth apps could serve as an important low-threshold entry point to these topics^63,64^.

### Limitations

Some limitations must be considered when interpreting the findings of this study. First, we only searched MHA available in European app stores in English or German language. Therefore, the findings cannot be generalized to apps available in other countries or languages. Second, due to the fast-paced nature of MHA development, it is conceivable that the content of some MHA may change or be no longer available on short to medium term^13^. Third, we used the MARS because it is a standard tool for systematic evaluation of MHA quality. Future studies could use different evaluating instruments which have other specific emphases, such as the APA app evaluation framework^65^ or the ENLIGHT tool^66^. Fourth, we only assessed privacy and security on a descriptive level. An assessment of privacy and security practice in smoking cessation and depression MHA with a more elaborated procedure showed that present privacy policies often lack adequate and sufficient information^67^. Future studies regarding MHA for diabetes could build on this procedure and assess whether data protection is still guaranteed under attack.

## Conclusions

At present, the potential of PSD principles implementation in diabetes self-management MHA may not be exploited optimally. PSD principles predict quality ratings of MHA and may play a crucial role in improving the engagement with MHA. Therefore, improvements in the implementation of PSD features in MHA should be achieved. Future studies should evaluate the engagement of real users with specific PSD principles to determine which features and functions work best in real life and thus contribute to the targeted improvement of diabetes self-management MHA. Finally, this study demonstrates an on average moderate quality of MHA with educational content targeting diabetes self-management available in European app stores, with deficits in information quality and engagement. As the more important it seems to establish databases such as mhad.science, psyberguide.org or mindapps.org in order to inform patients and healthcare providers about quality proven MHA in the vast quantity of available but most often not recommendable MHA for diabetes and beyond.

## Supplementary Information

Supplementary Table 1.

Supplementary Table 2.

Supplementary Table 3.

## Data Availability

All data generated or analyzed during this study are included in this published article (and its Supplementary Information files).
