# Supplementary material for: Standardized evaluation of the quality and persuasiveness of mobile health applications for diabetes management
Source: Sci Rep. 2022 Mar 7;12:3639. doi: 10.1038/s41598-022-07544-2 (PMC8901695; doi:10.1038/s41598-022-07544-2)
Supplement: Supplementary file 1 — Supplementary Table 1. [file 41598_2022_7544_MOESM1_ESM.docx]

|  |  |  | **Quality rating** | | | | | **Additional subscales** | | | |
| --- | --- | --- | --- | --- | --- | --- | --- | --- | --- | --- | --- |
| **Name** | **Developer** | **Total score** | **Engagement** | **Functionality** | **Aesthetics** | | **Information Quality** | **Therapeutic Gain** | **Subjective Quality** | | **Perceived Impact** |
| **Rated in Google Play Store** | | | | | | | | | | | |
| BD Diabetes Care App | BD - Digital Health | 4.01 | 4.20 | 4.38 | | 4.67 | 2.79 | 2.25 | 3.75 | 2.50 | |
| Hypo Program | Diabetes Digital Media | 3.90 | 3.80 | 4.38 | | 4.34 | 3.08 | 2.13 | 3.38 | 2.42 | |
| Jerry the Bear | Sproutel. Inc. | 3.88 | 3.80 | 4.25 | | 4.67 | 2.79 | 2.00 | 3.13 | 2.25 | |
| GLUCOSEZONE | Fitscript | 3.82 | 4.40 | 3.75 | | 4.00 | 3.15 | 2.38 | 3.75 | 3.58 | |
| Diabetes Words | LES LABORATOIRES SERVIER | 3.74 | 3.30 | 4.38 | | 4.84 | 2.43 | 2.13 | 3.38 | 2.50 | |
| DiabTrend - Diabetes Assistant | DiabTrend AI Analytics Kft. | 3.71 | 4.10 | 4.25 | | 4.00 | 2.50 | 2.50 | 3.50 | 3.25 | |
| My DiabetesConnect | Dr Ryzian Nizar MD MRCPUK | 3.65 | 3.60 | 4.38 | | 3.84 | 2.79 | 2.13 | 3.00 | 3.42 | |
| Glucose Control | Lehreer | 3.64 | 3.30 | 4.38 | | 4.17 | 2.71 | 2.25 | 3.63 | 2.50 | |
| Diabetes Forum | Diabetes Digital Media | 3.61 | 3.60 | 4.25 | | 4.00 | 2.57 | 1.75 | 3.25 | 2.08 | |
| Beyond Type 1 Diabetes | Mighty Networks | 3.55 | 3.60 | 4.13 | | 4.33 | 2.15 | 1.63 | 3.25 | 1.84 | |
| Life with Diabetes | Kosmograd | 3.53 | 3.20 | 4.13 | | 3.84 | 2.93 | 2.25 | 3.38 | 2.25 | |
| Intellin Diabetes Manager. Log & Risk Tracker | Intellin | 3.52 | 3.70 | 4.00 | | 3.67 | 2.71 | 2.25 | 3.13 | 2.84 | |
| Diafriendie - Best Friend for Diabetes | Karan Gill | 3.50 | 3.40 | 4.13 | | 4.17 | 2.29 | 1.75 | 2.38 | 1.75 | |
| Sugar Control - Diabetes management | Karim Timer | 3.49 | 3.50 | 4.38 | | 4.00 | 2.07 | 2.00 | 2.88 | 1.59 | |
| Diabetes | Focus Medica India Pvt. Ltd | 3.44 | 2.90 | 4.50 | | 4.00 | 2.36 | 1.88 | 2.25 | 2.25 | |
| Beat Diabetes | Tipsbook | 3.41 | 2.80 | 4.25 | | 4.17 | 2.43 | 2.00 | 3.00 | 2.00 | |
| Diabetes - Diabetes Diet Tips | FinalApps | 3.40 | 2.60 | 4.25 | | 4.17 | 2.57 | 2.13 | 3.25 | 2.25 | |
| Diabetes Hub - Diabetics foods. Tips & and Forum | DONG HUANHUAN | 3.39 | 3.20 | 4.00 | | 4.00 | 2.36 | 1.88 | 2.38 | 1.67 | |
| DIABETES TREATMENT App | Vission Assist | 3.33 | 2.50 | 4.25 | | 4.00 | 2.57 | 2.13 | 3.00 | 2.17 | |
| Help My Diabetes | Healthcare app | 3.33 | 3.10 | 4.00 | | 3.84 | 2.36 | 1.88 | 3.00 | 2.00 | |
| Type 2 diabetes | Focus Medica India Pvt. Ltd | 3.33 | 2.60 | 4.38 | | 4.00 | 2.36 | 1.88 | 2.00 | 2.17 | |
|  |  |  |  |  | |  |  |  |  |  | |
| Myabetic Diabetes TV | Myabetic LLC | 3.31 | 2.60 | 4.25 | | 4.17 | 2.22 | 2.00 | 2.75 | 1.84 | |
| BEAT Diabetes & Prediabetes | Virtual Health SHED Ltd. | 3.30 | 2.50 | 4.13 | | 4.00 | 2.57 | 2.00 | 2.50 | 2.09 | |
| 7 Day Diabetic Meal Plan | Matanopp | 3.29 | 2.40 | 4.25 | | 4.00 | 2.50 | 1.88 | 2.63 | 1.83 | |
| Diabetes Diet | Investment Tips | 3.28 | 2.70 | 4.13 | | 4.00 | 2.29 | 1.88 | 2.50 | 2.00 | |
| Diabetic Diet | Gato Apps | 3.28 | 2.60 | 3.88 | | 4.00 | 2.64 | 2.13 | 2.75 | 2.09 | |
| Diabetic Diet Plan( Diabetic Diet Information) | TechMono360 | 3.26 | 2.70 | 4.25 | | 4.00 | 2.07 | 2.00 | 3.00 | 2.17 | |
| Beat Diabetes Pro - Ad Free | Tipsbook | 3.25 | 2.80 | 3.88 | | 3.67 | 2.64 | 2.00 | 2.50 | 3.33 | |
| Diabetes Care | Startup Media | 3.25 | 2.60 | 4.38 | | 4.17 | 1.86 | 2.00 | 2.75 | 2.17 | |
| Diabetic Diet Plan | Appland Studios | 3.25 | 2.60 | 4.25 | | 4.00 | 2.15 | 2.13 | 3.13 | 2.25 | |
| Diabetic diet | AbacaxiApps | 3.24 | 2.60 | 4.50 | | 4.00 | 1.86 | 2.13 | 2.75 | 2.17 | |
| Pregnant with diabetes | heyworld.dk | 3.24 | 2.30 | 4.25 | | 4.17 | 2.22 | 2.00 | 3.13 | 2.25 | |
| All About Diabetes - A Complete Diabetes Guide | Beracah | 3.22 | 2.50 | 3.88 | | 3.84 | 2.64 | 2.00 | 3.13 | 2.25 | |
| Diabetes Care Diet and Nutrition | Data Recovery Software by RecoveryBull.com | 3.22 | 3.00 | 4.13 | | 3.84 | 1.93 | 1.75 | 2.13 | 3.75 | |
| diabetes | muntaser ineim | 3.21 | 2.50 | 4.25 | | 3.67 | 2.43 | 2.25 | 3.00 | 2.42 | |
| Diabetes Varnamala | Vineet L Rao | 3.19 | 2.80 | 4.38 | | 3.84 | 1.72 | 1.75 | 2.25 | 1.75 | |
| Diabetes Yoga Therapy at Home - Diet for low Sugar | Dr. Zio - Yoga Teacher | 3.19 | 3.40 | 3.75 | | 3.17 | 2.43 | 1.75 | 2.00 | 2.50 | |
| Blood Sugar Diary. Blood Glucose Tracker | StudySpring | 3.18 | 3.00 | 4.13 | | 3.67 | 1.93 | 2.00 | 2.63 | 1.67 | |
| Diabetes and Obesity | CVDN | 3.18 | 2.60 | 4.25 | | 4.00 | 1.86 | 2.00 | 2.50 | 2.17 | |
| Signs & Symptoms Diabetic Foot | Built by Doctors World Ltd | 3.18 | 3.10 | 4.00 | | 3.17 | 2.43 | 2.00 | 2.25 | 2.00 | |
| Diabetic Diet Plan | Chiquito Apps | 3.17 | 2.30 | 3.88 | | 4.00 | 2.50 | 1.88 | 2.75 | 1.92 | |
| Type 2 Diabetes Healthy Eating | Deeswad | 3.16 | 2.30 | 4.25 | | 3.50 | 2.57 | 2.00 | 2.63 | 1.92 | |
| Diabetes Control | Dr_Apps | 3.15 | 2.50 | 4.38 | | 3.50 | 2.22 | 2.00 | 2.88 | 1.92 | |
| Type 2 Diabetes | ZaidHBB | 3.15 | 2.40 | 4.13 | | 4.00 | 2.07 | 2.00 | 2.75 | 2.25 | |
| Wie man Diabetes behandelt | goGOODapp | 3.14 | 2.20 | 4.50 | | 3.84 | 2.00 | 2.00 | 2.50 | 2.00 | |
| Diabetes Care | Master Apps - Fashion. Lifestyle. Recipes | 3.13 | 2.30 | 4.13 | | 4.00 | 2.07 | 1.38 | 1.88 | 1.59 | |
| Diabetes Type 1 | rnamobile | 3.13 | 2.40 | 4.38 | | 3.67 | 2.07 | 2.00 | 2.75 | 2.00 | |
| Signs & Symptoms Diabetes | Built by Doctors World Ltd | 3.11 | 3.20 | 3.63 | | 3.17 | 2.43 | 2.00 | 2.38 | 2.08 | |
| Diabetes: Type 1&2 | Ysfmamman | 3.10 | 2.40 | 4.25 | | 3.67 | 2.07 | 2.13 | 2.63 | 1.75 | |
| Blood Sugar Diet - Sugar Checker - Sugar Info | BS Soultions | 3.09 | 2.30 | 4.25 | | 3.50 | 2.29 | 2.00 | 2.00 | 1.67 | |
| Diabetes Care | Kalpesh Lakhani | 3.09 | 2.60 | 4.38 | | 3.67 | 1.72 | 1.75 | 2.38 | 1.67 | |
| Blood Sugar Levels (A to Z) | Appmax365 | 3.08 | 1.90 | 4.13 | | 3.84 | 2.43 | 2.00 | 2.50 | 1.84 | |
| Diabetic Diet Plan Chart | Solution Maker | 3.08 | 2.60 | 3.88 | | 3.33 | 2.50 | 2.00 | 2.75 | 2.17 | |
| Easy Diabetic Recipes | Leh | 3.06 | 2.80 | 3.75 | | 3.67 | 2.00 | 1.75 | 2.63 | 1.92 | |
| Diabetes Symptoms Causes | High Soft App | 3.04 | 2.20 | 4.38 | | 3.00 | 2.57 | 2.00 | 2.63 | 1.92 | |
| Diabetic Diet Plan Chart | Appmax365 | 3.03 | 2.20 | 4.13 | | 3.50 | 2.29 | 2.00 | 2.75 | 2.00 | |
| Diabetic Protocols | Dr.Isaac's Holistic Wellness | 3.02 | 2.20 | 3.88 | | 3.50 | 2.50 | 1.75 | 2.13 | 1.84 | |
| Home Remedies For Diabetes | RK Unit | 3.00 | 2.20 | 4.13 | | 3.67 | 2.00 | 1.75 | 2.13 | 1.75 | |
| Type 2 Diabetes | ARUNAS APPS LLP | 3.00 | 2.30 | 4.38 | | 3.17 | 2.15 | 2.00 | 2.63 | 2.00 | |
| Control Your Diabetes | freeCreativity2019 | 2.99 | 2.20 | 4.25 | | 3.50 | 2.00 | 2.00 | 2.88 | 2.00 | |
| gestational diabetes | Health Care Tips | 2.99 | 2.40 | 4.25 | | 3.50 | 1.79 | 2.00 | 2.75 | 2.00 | |
| Type 2 Diabetes Healthy Eating | salim garba usman | 2.97 | 2.30 | 4.25 | | 3.33 | 2.00 | 2.00 | 2.50 | 2.00 | |
| Diabetes Diet and Management | GangareBoy | 2.96 | 2.20 | 4.25 | | 3.33 | 2.07 | 2.00 | 2.50 | 2.00 | |
| Type 2 Diabetes Diet Plan | BrotherHoodApp | 2.95 | 2.30 | 4.00 | | 3.50 | 2.00 | 2.00 | 2.63 | 2.09 | |
| Diabetes | A to Z Cure | 2.94 | 2.10 | 4.25 | | 3.33 | 2.08 | 1.50 | 1.88 | 1.50 | |
| Diabetic Diet & Symptoms of diabetes - Diabetes Go | LazerApps Inc. | 2.94 | 2.20 | 4.00 | | 3.50 | 2.07 | 1.88 | 2.38 | 1.75 | |
| Foods to Avoid with Diabetes | The Future Dev | 2.94 | 2.20 | 4.25 | | 3.50 | 1.79 | 1.88 | 2.63 | 1.92 | |
| Blood Sugar Levels - Knowledge | Vission Assist | 2.93 | 2.20 | 3.63 | | 3.67 | 2.22 | 2.00 | 2.50 | 2.17 | |
| Diabetes Fitness | Ankit Chauhan | 2.93 | 2.70 | 3.75 | | 3.17 | 2.08 | 1.75 | 2.13 | 1.50 | |
| glucose levels | EL MAKAOUI | 2.93 | 2.20 | 4.13 | | 3.17 | 2.22 | 1.88 | 2.13 | 1.67 | |
| Is your Diabetes under control | Twayesh Projects | 2.93 | 2.10 | 4.25 | | 3.50 | 1.86 | 2.00 | 2.25 | 1.84 | |
| schlagen Diabetes | DEVAPPSINC | 2.91 | 2.20 | 3.88 | | 3.50 | 2.07 | 1.88 | 2.50 | 1.92 | |
| diabetes | Health Care Tips | 2.88 | 2.20 | 4.13 | | 3.17 | 2.00 | 1.75 | 1.75 | 1.59 | |
| Diabetes Mellitus | Kadira Apps | 2.88 | 2.00 | 4.38 | | 3.33 | 1.79 | 1.88 | 2.00 | 1.59 | |
| Blood Sugar Test + Info and Advice | Olivia saint luise | 2.87 | 2.70 | 4.00 | | 2.84 | 1.93 | 2.00 | 2.00 | 1.75 | |
| Diabetes Guide | Smartcookie - Protsahanbharati | 2.86 | 2.20 | 4.13 | | 3.33 | 1.79 | 1.88 | 2.13 | 1.92 | |
| Hyperglykämie | EL MAKAOUI | 2.85 | 2.20 | 4.25 | | 3.17 | 1.79 | 1.75 | 2.13 | 1.67 | |
| Diabetes : How to Controle Diabetes. Diabetes Diet | Vdicts | 2.83 | 2.00 | 4.38 | | 3.50 | 1.43 | 1.50 | 1.63 | 1.50 | |
| Diabetes symptoms | FabGiver Apps | 2.81 | 2.20 | 4.13 | | 3.33 | 1.57 | 2.00 | 2.25 | 1.67 | |
| Diabetes Treatment | Xtell Technologies | 2.80 | 2.10 | 4.25 | | 3.00 | 1.86 | 1.75 | 1.88 | 1.59 | |
| Diabetes App | Z_T Gurmani | 2.77 | 2.20 | 4.25 | | 2.84 | 1.79 | 1.75 | 1.75 | 1.75 | |
| Diabetes Control Tips | Dudly World | 2.75 | 2.40 | 4.00 | | 3.17 | 1.43 | 1.63 | 1.63 | 1.42 | |
| Diabetes type 1 and 2 Cure Check Up Monitor App | Beatrix | 2.69 | 2.00 | 3.88 | | 3.17 | 1.72 | 1.38 | 1.75 | 1.59 | |
| Diabetes – Blood Sugar | Blood Sugar app | 2.65 | 2.10 | 3.75 | | 3.33 | 1.43 | 1.75 | 1.88 | 1.59 | |
| Herbal Concoctions For Diabetes | oemahwangi | 2.58 | 2.00 | 4.00 | | 2.67 | 1.65 | 1.25 | 1.38 | 1.17 | |
| Diabetes | Utkarsh_kumar | 2.57 | 2.10 | 3.75 | | 2.34 | 2.07 | 1.75 | 1.75 | 1.67 | |
| Blood Sugar Calculator. Info. Dairy. Log History | World Softech Apps | 2.52 | 2.50 | 3.13 | | 2.67 | 1.79 | 1.63 | 1.75 | 1.42 | |
| Diabetes | Flash 4gexLasting | 2.32 | 1.90 | 3.13 | | 3.17 | 1.07 | 1.50 | 1.63 | 1.25 | |
| Blood Sugar Test Converter and Info | QConnectappstore | 2.31 | 1.80 | 3.38 | | 2.84 | 1.22 | 1.38 | 1.50 | 1.50 | |
| **Rated in Apple iTunes Store** | | | | | | | | | | | |
| X-PERT | Pulse Digital | 4.61 | 4.60 | 4.50 | | 4.84 | 4.50 | 4.13 | 4.38 | 4.67 | |
| One Drop Diabetes Management | Informed Data Systems. Inc. | 3.93 | 4.00 | 4.38 | | 4.00 | 3.36 | 2.88 | 3.50 | 3.75 | |
| Dario | LabStyle Innovation Ltd | 3.78 | 4.00 | 4.25 | | 4.17 | 2.72 | 3.13 | 3.13 | 3.42 | |
| Diabetes Tracker by MyNetDiary | MyNetDiary Inc. | 3.70 | 3.80 | 4.13 | | 4.17 | 2.72 | 2.25 | 2.75 | 3.25 | |
| iHealth Gluco Smart | iHealth Labs Inc. | 3.67 | 3.90 | 4.00 | | 3.84 | 2.93 | 2.38 | 3.50 | 3.08 | |
| Diabetes App: BD Diabetes Care | Becton. Dickinson. and Company | 3.62 | 3.60 | 4.25 | | 4.00 | 2.64 | 2.25 | 3.50 | 3.67 | |
| DMP | TLC Platforms Inc. | 3.61 | 4.00 | 4.00 | | 4.17 | 2.29 | 1.88 | 3.50 | 3.09 | |
| DDH-M Digital | Deutsche Diabetes Hilfe- Menschen mit Diabetes DDH-M e.V. | 3.54 | 3.40 | 4.25 | | 4.00 | 2.50 | 2.13 | 3.50 | 3.42 | |
| GlucoSecrets | Shunzhe Ma | 3.50 | 3.30 | 4.25 | | 4.00 | 2.43 | 2.38 | 3.00 | 3.25 | |
| Kids and Teens Diabetes | A. Menarini Diagnostics S.r.l. | 3.50 | 3.10 | 4.50 | | 3.83 | 2.57 | 2.25 | 2.88 | 3.42 | |
| Diabetes Health Manager | @ Point of care | 3.41 | 3.60 | 3.88 | | 3.67 | 2.50 | 2.13 | 2.63 | 3.00 | |
| Dia Aid | Zone35 GmbH & Co. KG | 3.38 | 3.50 | 4.38 | | 3.50 | 2.15 | 2.00 | 2.63 | 2.08 | |
| Our Journey with Diabetes | Phoenix Children's Hospital. Inc | 3.35 | 3.20 | 4.50 | | 3.50 | 2.22 | 2.38 | 2.75 | 3.67 | |
| Diabetes Low Carb Diet Apps | DigitalMarketer | 3.30 | 3.30 | 4.00 | | 3.67 | 2.22 | 1.63 | 2.00 | 2.42 | |
| Diabetes Pass App | Schweizerische Diabetes-Stiftung | 3.28 | 3.30 | 4.25 | | 3.67 | 1.92 | 2.25 | 2.75 | 2.25 | |
| RapidCalc Diabetes Manager | Gilport Enterpirses | 3.20 | 3.10 | 3.50 | | 3.33 | 2.86 | 2.75 | 2.00 | 2.09 | |
| iFORA Diabetes Manager | ForaCare Inc. | 3.16 | 2.90 | 3.88 | | 3.17 | 2.72 | 2.00 | 2.25 | 2.34 | |
| Diabetes Clinical Care | Börm Bruckmeier Publishing LLC | 3.08 | 2.50 | 4.00 | | 3.17 | 2.65 | 2.00 | 3.00 | 2.59 | |
| URIGHT Diabetes Manager | TaiDoc | 3.05 | 3.00 | 3.88 | | 2.84 | 2.50 | 2.00 | 2.25 | 2.50 | |
| Diabetic Diet Plan: Guide and Recipes | Diego Correa Bonini | 2.99 | 2.80 | 3.50 | | 3.17 | 2.50 | 2.00 | 2.38 | 2.92 | |
| KE-Finder | Cougar Media & Analysis GmbH | 2.99 | 2.80 | 4.13 | | 2.84 | 2.22 | 1.88 | 2.25 | 1.50 | |
| Glukometer: Diabetes-Tracking | Onur Yuzbasioglu | 2.95 | 2.40 | 4.13 | | 3.50 | 1.79 | 1.63 | 1.63 | 2.17 | |
| GluceoPro Diabetes Manager | METRADO GmbH | 2.94 | 2.60 | 4.00 | | 2.67 | 2.50 | 2.00 | 1.63 | 2.00 | |
| MEET ME @ 7 - Diabetes Self-management Tool for Patients and Caregivers | AtlantiCare | 2.91 | 2.60 | 3.88 | | 3.17 | 2.00 | 1.88 | 2.00 | 2.42 | |
| diab2gether Diabetes Community | DBW Diabetiker Baden Württemberg e.V. | 2.73 | 2.80 | 3.25 | | 3.17 | 1.72 | 1.50 | 1.75 | 1.84 | |
| DiaBeatMove-Meal. CGM. Insulin | Karazel Balance Inc. | 2.73 | 2.80 | 3.13 | | 3.00 | 2.00 | 1.63 | 1.38 | 1.75 | |
| Sugar Control Diary | 晓宁 冼 | 2.69 | 2.40 | 3.38 | | 2.84 | 2.14 | 1.63 | 1.63 | 1.75 | |
| Broteinheiten | Cougar Media & Analysis GmbH | 2.64 | 2.10 | 3.75 | | 3.00 | 1.72 | 1.75 | 2.13 | 1.67 | |
| **Rated in both app stores** | | | | | | | | | | | |
| myDiabetes | My mhealth | 4.62 | 4.80 | 4.75 | | 5.00 | 3.93 | 4.75 | 4.38 | 4.58 | |
| Invincible | Invincible Corp. | 4.02 | 4.30 | 4.38 | | 4.84 | 2.58 | 2.25 | 3.63 | 3.75 | |
| Glucose Buddy Diabetes Tracker | Azumio Inc. | 3.95 | 4.3 | 4.25 | | 4.17 | 3.07 | 2.50 | 3.88 | 3.92 | |
| **Total mean (SD)** |  | **3.20**  **(0.39)** | **2.80**  **(0.67)** | **4.10**  **(0.30)** | | **3.64**  **(0.50)** | **2.26**  **(0.48)** | **2.00**  **(0.42)** | **2.58**  **(0.62)** | **2.23**  **(0.69)** | |

*Note.* Engagement = Five items on fun, interest, individual adaptability, interactivity, target group; Functionality = Four items on performance, usability, navigation, gestural design; Aesthetics = Three items on layout, graphics, visual appeal; Information quality = Seven items on accuracy of app description, goals, quality of information, quantity of information, quality of visual information, credibility, evidence base; Therapeutic gain = Four items on gain for patients, gain for therapists, risks and side effects, ease of implementation into routine healthcare; Subjective quality = Four items on recommendation, frequency of use, willingness to pay, overall star rating; Perceived impact = Six items on awareness, knowledge, attitudes, intention to change, help seeking, behavioral change; Total score = Mean of sore of the engagement, functionality, aesthetics, information quality subscales
