# Supplementary material for: Standardized evaluation of the quality and persuasiveness of mobile health applications for diabetes management
Source: Sci Rep. 2022 Mar 7;12:3639. doi: 10.1038/s41598-022-07544-2 (PMC8901695; doi:10.1038/s41598-022-07544-2)
Supplement: Supplementary file 3 — Supplementary Table 3. [file 41598_2022_7544_MOESM3_ESM.docx]

| **Name** | **Developer** | **MARS Score** | **Diabetes pathophysiology and treatment options** | **Healthy nutrition** | **Physical activity** | **Medication usage** | **Monitoring and using patient-generated health data** | **Preventing, detecting, and treating acute and chronic complications** | **Healthy coping with psychosocial issues and concerns** | **Problem solving** |
| --- | --- | --- | --- | --- | --- | --- | --- | --- | --- | --- |
| myDiabetes | my mhealth | 4,62 | x | x | x | x | x | x | x |  |
| X-PERT Diabetes Digital | Pulse Digital | 4,61 | x | x | x | x | x | x | x | x |
| Invincibile | Invincible Corp. | 4,02 | x |  |  |  |  |  | x | x |
| BD Diabetes Care App | BD - Digital Health | 4,01 |  | x | x | x | x |  |  |  |
| Glucose Buddy Diabetes Tracker | Azumio, Inc. | 3,95 | x | x | x | x | x | x | x |  |
| One Drop Diabetes Management | Informed Data Systems, Inc. | 3,93 |  |  |  |  | x |  | x |  |
| Hypo Program | Diabetes Digital Media | 3,9 | x | x | x |  | x | x | x | x |
| Jerry the Bear | Sproutel, Inc. | 3,88 | x |  |  |  |  |  |  |  |
| GLUCOSEZONE | Fitscript | 3,82 |  |  | x |  |  |  |  |  |
| Dario | LabStyle Innovation Ltd | 3,78 | x |  |  |  |  |  | x | x |
| Diabetes Words | LES LABORATOIRES SERVIER | 3,74 | x | x | x |  |  | x | x | x |
| DiabTrend - Diabetes Assistant | DiabTrend AI Analytics Kft. | 3,71 | x | x | x | x | x | x | x | x |
| Diabetes Tracker by MyNetDiary | MyNetDiary Inc. | 3,70 |  | x |  | x | x |  |  |  |
| iHealth Gluco-Smart | iHealth Labs Inc. | 3,67 | x |  |  |  | x |  | x | x |
| My DiabetesConnect | Dr Ryizan Nizar MD MRCPUK | 3,65 | x | x |  |  | x | x |  |  |
| Glucose Control | Lehreer | 3,64 | x | x |  |  | x | x | x |  |
| Diabetes App: BD Diabetes Care | Becton, Dickinson, and Company | 3,62 | x | x | x |  | x | x | x |  |
| Diabetes Forum | Diabetes Digital Media | 3,61 | x |  |  |  |  |  |  |  |
| DMP | TLC Platforms Inc. | 3,61 | x | x | x | x | x | x | x | x |
| Beyond Type 1 Diabetes | Mighty Networks | 3,55 |  |  |  |  |  |  |  |  |
| DDH-M Digital | Deutsche Diabetes-Hilfe - Menschen mit Diabetes DDH-M e.V. | 3,54 | x | x | x | x | x | x | x | x |
| Life with Diabetes | Kosmograd | 3,53 | x | x | x |  | x |  | x | x |
| Intellin Diabetes Manager, Log & Risk Tracker | Intellin | 3,52 |  | x | x |  | x | x |  |  |
| Diafriendie - Best Friend for Diabetes | Karan Gill | 3,5 | x | x | x |  |  | x | x | x |
| GlucoSecrets | Shunzhe Ma | 3,50 |  |  | x |  | x |  |  |  |
| Kids and Teens Diabetes | A. Menarini Diagnostics S.r.l. | 3,50 | x |  | x |  | x | x | x | x |
| Sugar Control - Diabetes management | Karim Timer | 3,49 | x |  |  |  | x | x |  | x |
| Diabetes | Focus Medica India Pvt. Ltd | 3,44 | x |  |  |  |  | x |  |  |
| Beat Diabetes | Tipsbook | 3,41 | x | x | x |  |  | x | x |  |
| Diabetes Health Manager | @Point of Care | 3,41 | x | x | x | x | x | x | x | x |
| Diabetes - Diabetes Diet Tips | FinalApps | 3,4 | x | x | x | x |  | x | x |  |
| Diabetes Hub - Diabetics foods, Tips & and Forum | DONG HUANHUAN | 3,39 | x | x |  |  |  |  |  | x |
| Dia Aid | Zone35 GmbH & Co. KG | 3,38 | x | x | x | x | x | x | x | x |
| Our Journey with Diabetes | Phoenix Children's Hospital, Inc | 3,35 | x |  |  |  | x |  | x | x |
| DIABETES TREATMENT App | Vission Assist | 3,33 | x | x | x | x | x | x |  | x |
| Type 2 diabetes | Focus Medica India Pvt. Ltd | 3,33 | x |  |  |  |  | x |  |  |
| Help my diabetes | Healthcare app | 3,32 | x | x | x | x | x | x |  | x |
| Myabetic Diabetes TV | Myabetic LLC | 3,31 | x | x | x |  |  |  |  |  |
| BEAT Diabetes & Prediabetes | Virtual Health SHED Ltd. | 3,3 | x | x | x | x | x | x |  |  |
| Diabetes Low Carb Diet Apps | DigitalMarketer | 3,30 |  |  |  |  |  |  |  |  |
| 7 Day Diabetic Meal Plan | Matanopp | 3,29 |  | x |  |  |  |  |  |  |
| Diaebtic Diet | Gato Apps | 3,28 | x | x | x |  |  | x | x |  |
| Diabetes Diet | Investment Tips | 3,28 |  | x | x |  |  | x | x |  |
| Diabetes Pass App | Schweizerische Diabetes-Stiftung | 3,28 |  |  | x |  | x | x |  | x |
| Diabetic Diet Plan( Diabetic Diet Information) | TechMono360 | 3,26 | x | x |  |  |  |  |  |  |
| Diabetes Care | Startup Media | 3,25 | x | x | x |  | x | x |  |  |
| Diabetic Diet Plan | Appland Studios | 3,25 | x | x | x | x | x | x | x |  |
| Beat Diabetes Pro - Ad Free |  | 3,25 | x | x | x |  |  | x | x |  |
| Diabetic diet | AbacaxiApps | 3,24 | x | x | x |  |  | x | x |  |
| Pregnant with diabetes | heyworld.dk | 3,24 | x | x | x | x | x | x |  | x |
| All About Diabetes - A Complete Diabetes Guide | Beracah | 3,22 | x | x | x | x | x | x | x |  |
| Diabetes Care Diet & Nutrition | Data Recovery Software by RecoveryBull.com | 3,22 | x | x | x |  |  | x |  | x |
| diabetes | muntaser ineim | 3,21 | x | x | x | x | x | x | x |  |
| RapidCalc Diabetes Manager | Gilport Enterprises | 3,20 |  |  |  |  | x |  |  |  |
| Diabetes Varnamala | Vineet L Rao | 3,19 | x | x | x |  | x | x | x | x |
| Diabetes Yoga Therapy at Home - Diet for low Sugar | Dr. Zio - Yoga Teacher | 3,19 | x | x | x |  |  |  |  |  |
| Signs & Symptoms Diabetic Foot | Built by Doctors World Ltd | 3,18 |  |  |  |  |  | x |  |  |
| Diabetes and Obesity | CVDN | 3,18 | x | x | x |  |  | x | x |  |
| Blood Sugar Diary, Blood Glucose Tracker | StudySpring | 3,18 | x | x | x | x | x |  |  |  |
| Diabetic Diet Plan | Chiquito Apps | 3,17 |  | x | x |  |  |  |  |  |
| Type 2 Diabetes Healthy Eating | Deeswad | 3,16 | x | x | x | x | x |  |  | x |
| iFORA Diabetes Manager | ForaCare Inc. | 3,16 | x | x | x | x | x |  |  |  |
| Diabetes Control | Dr_Apps | 3,15 | x | x | x | x | x | x | x |  |
| Type 2 Diabetes | ZaidHBB | 3,15 | x | x |  | x |  |  |  |  |
| Wie man Diabetes behandelt | goGOODapp | 3,14 | x | x | x | x | x |  |  |  |
| Diabetes Type 1 | rnamobile | 3,13 | x | x | x | x |  | x | x | x |
| Diabetes Care | Master Apps - Fashion, Lifestyle, Recipes | 3,13 | x | x | x |  |  |  |  |  |
| Signs & Symptoms Diabetes | Built by Doctors World Ltd | 3,11 | x |  |  |  | x | x |  |  |
| Diabetes: Type 1&2 | Ysfmamman | 3,1 | x | x | x |  |  |  |  |  |
| Diabetes Care | Kalpesh Lakhani | 3,09 | x | x |  |  | x | x | x |  |
| Blood Sugar Diet - Sugar Checker - Sugar Info | BS Soultions | 3,09 | x | x |  |  |  | x |  |  |
| Diaebetic Diet Plan Chart | Solution Maker | 3,08 |  | x |  |  |  |  |  |  |
| Blood Sugar Levels (A to Z) | Appmax365 | 3,08 |  | x | x |  | x |  |  | x |
| Diabetes Clinical Care | Börm Bruckmeier Publishing LLC | 3,08 | x |  |  |  | x | x |  |  |
| Easy Diabetic Recipes | Leh | 3,06 |  | x |  |  |  |  |  |  |
| URIGHT Diabetes Manager | TaiDoc | 3,05 | x | x | x | x | x |  |  |  |
| Diabetes Symptoms Causes | High Soft App | 3,04 | x | x | x | x | x | x |  |  |
| Diabetic Diet Plan Chart | Appmax365 | 3,03 |  | x | x |  |  | x |  |  |
| Diabetic Protocols | Dr.Isaac's Holistic Wellness | 3,02 | x | x | x |  |  |  |  |  |
| Type 2 Diabetes | ARUNAS APPS LLP | 3 | x | x | x | x |  |  |  |  |
| Home Remedies For Diabetes | RK Unit | 3 | x | x | x |  | x |  |  |  |
| gestational diabetes | Health Care Tips | 2,99 | x | x |  | x | x |  | x |  |
| Control Your Diabetes | freeCreativity2019 | 2,99 | x | x | x | x | x | x | x |  |
| KE-Finder | Cougar Media & Analysis GmbH | 2,99 | x (news) | x |  |  |  |  |  |  |
| Diabetic Diet Plan: Guide and Recipes | Diego Correa Bonini | 2,99 | x | x | x |  |  |  |  |  |
| Type 2 Diabetes Healthy Eating | salim garba usman | 2,97 | x | x | x | x | x | x |  | x |
| Diabetes Diet and Management | GangareBoy | 2,96 | x | x | x |  | x | x |  | x |
| Type 2 Diabetes Diet Plan | BrotherHoodApp | 2,95 | x | x |  |  |  |  |  |  |
| Glukometer: Diabetes-Tracking | Onur Yuzbasioglu | 2,95 | x | x | x | x | x | x |  |  |
| Diabetic Diet & Symptoms of diabetes - Diabetes Go | LazerApps Inc. | 2,94 | x | x | x | x | x |  |  |  |
| Diabetes | A to Z Cure | 2,94 | x | x | x |  |  |  |  |  |
| Foods to Avoid with Diabetes | The Future Dev | 2,94 | x | x | x |  |  | x |  |  |
| GluceoPro Diabetes Manager | METRADO GmbH | 2,94 | x | x | x | x | x |  |  |  |
| Diabetes Fitness | Ankit Chauhab | 2,93 |  | x | x |  | x |  |  |  |
| glucose levels | EL MAKAOUI | 2,93 |  |  |  |  | x | x |  |  |
| Is your Diabetes under control | Twayesh Projects | 2,93 | x | x | x | x | x | x | x |  |
| Blood Sugar Levels - Knowledge | Vission Assist | 2,93 | x | x | x |  | x | x | x |  |
| schlagen Diabetes | DEVAPPSINC | 2,91 | x | x | x | x | x |  | x |  |
| MEET ME @7 - Diabetes Self-management Tool for Patients and Caregivers | AtlantiCare | 2,91 |  | x | x | x | x | x |  | x |
| Diabetes Mellitus | Kadira Apps | 2,88 | x | x | x | x |  | x |  |  |
| diabetes | Health Care Tips | 2,88 | x |  |  |  |  | x |  |  |
| Blood Sugar Test + Info and Advice | Olivia saint luise | 2,87 | x | x | x |  | x |  | x |  |
| Diabetes Guide | Smartcookie - Protsahanbharati | 2,86 | x | x | x | x |  | x |  |  |
| Hyperglykämie | EL MAKAOUI | 2,85 | x |  |  |  | x | x |  |  |
| How to Controle Diabetes, Diabetes Diet | Vdicts | 2,83 | x | x |  |  |  | x |  |  |
| Diabetes symptoms | FabGiver Apps | 2,81 | x | x | x | x |  | x |  |  |
| Diabetes treatment | Xtell Technologies | 2,8 | x | x | x | x | x | x |  |  |
| Diabetes App | Z_T Gurmani | 2,77 | x | x |  |  | x | x |  | x |
| Diabetes Control Tips | Dudly World | 2,75 |  | x | x |  |  |  | x |  |
| DiaBeatMove-Meal, CGM, Insulin | Karazel Balance Inc. | 2,73 |  |  |  |  | x |  |  |  |
| diab2gether Diabetes Community | DBW Diabetiker Baden-Württemberg e.V. | 2,73 | x | x | x | x | x | x | x | x |
| Diabetes type 1 and 2 Cure Check Up Monitor App | Beatrix | 2,69 | x | x | x | x | x | x |  |  |
| Sugar Control Diary | 晓宁 冼 | 2,69 |  |  |  | x | x | x |  |  |
| Diabetes - Blood Sugar | Blood Sugar app | 2,65 | x | x | x |  |  | x | x |  |
| Broteinheiten | Cougar Media & Analysis GmbH | 2,64 |  | x |  |  |  |  |  |  |
| Herbal Concoctions For Diabetes | oemahwangi | 2,58 |  | x |  |  |  |  |  |  |
| Diabetes | Utkarsh_kumar | 2,57 | x |  | x |  |  |  | x |  |
| Blood Sugar Calculator, Info, Dairy, Log History | World Softech Apps | 2,52 | x |  |  | x | x | x |  |  |
| Diabetes | Flash 4gexLasting | 2,32 | x |  |  |  |  |  |  |  |
| Blood Sugar Test Converter and Info | QConnectappstore | 2,31 |  | x | x |  |  | x |  |  |

*Note.* Self-management tasks according to the American Association of Diabetes Educators.
